# Neuroimaging findings of posterior reversible encephalopathy syndrome (PRES) following haematopoietic stem cell transplantation in paediatric recipients

**DOI:** 10.1186/s12887-021-02890-y

**Published:** 2021-10-11

**Authors:** Ali Önder Atça, Berrin Erok, Selime Aydoğdu

**Affiliations:** 1 Department of Radiology, Altınbas University School of Medicine Bahcelievler Medical Park Hospital, İstanbul, Turkey; 2 Department of Radiology, University of Health Sciences, Prof Dr Cemil Tascıoglu City Hospital, Istanbul, Turkey; 3 Department of Hematology, Altınbas University School of Medicine Bahcelievler Medical Park Hospital, İstanbul, Turkey

**Keywords:** Haematopoietic stem cell transplantation, Posterior reversible encephalopathy syndrome, CNS complications, PRES, MRI

## Abstract

**Background:**

Haematopoietic stem cell transplantation (HSCT) is used worldwide in various malignant and nonmalignant childhood diseases, including haematologic, genetic, autoimmune and metabolic disorders, and is the only curative treatment for many of these illnesses. The survival rates of many childhood diseases have been increased due to HSCT treatment. However, associated complications are still important for management. Central nervous system (CNS) complications in paediatric HSCT recipients can be associated with high morbidity and significantly contribute to mortality. Posterior reversible encephalopathy syndrome (PRES) is one of the most common CNS complications in patients with neurological symptoms following HSCT. Magnetic resonance imaging (MRI) is the modality of choice and shows typical bilateral vasogenic oedema at the posterior parts of the cerebral hemispheres; however, various atypical imaging manifestations can also occur. In this study, we retrospectively examined CNS complications in our paediatric HSCT recipients with a focus on the typical and atypical neuroimaging manifestations of PRES following HSCT.

**Methods:**

We retrospectively reviewed the medical records of 300 consecutive paediatric HSCT recipients from January 2014 to November 2018. A total of 130 paediatric HSCT recipients who experienced neurological signs and symptoms and were evaluated with neuroimaging studies following HSCT were enrolled in the study. The timing of CNS complications was defined according to immune status, including the pre-engraftment period (< 30 days after HSCT), the early postengraftment period (30–100 days after HSCT), and the late postengraftment period (> 100 days after HSCT), which were defined as phases 1, 2 and 3, respectively.

**Results:**

Overall, 130 paediatric HSCT recipients experienced neurological signs and symptoms and therefore underwent neuroimaging examinations. Among these 130 patients, CNS complications were present in 23 patients (17.6%, 23/130), including 13 (56.5%) females and 10 (43.5%) males with a median age of 8.0 years (range, 8 months to 18.0 years). Among these 23 patients, 14 cases of PRES (60.9%), 5 (21.7%) cases of leukoencephalopathy, 3 cases of acute subdural haemorrhage (ASDH) (13%) and 1 (4.3%) case of fungal CNS infection were identified by neuroimaging. On MRI, typical parietooccipital vasogenic oedema was present in 78.5% of the PRES cases (11/14). The following atypical neuroimaging manifestations were observed: isolated involvement of the bilateral frontal lobes in 1 case, isolated cerebellar vermis involvement in 1 case, and isolated basal ganglia involvement in 1 case. Restricted diffusion associated with cytotoxic damage was demonstrated in 2 of 14 cases, one of which also showed subacute cytotoxic injury with ADC pseudonormalization.

**Conclusion:**

Paediatric HSCT recipients presenting with CNS signs and symptoms should be evaluated by neuroimaging studies for timely diagnosis and early management. PRES is the most common CNS complication and may present with atypical MRI manifestations, which should not dissuade a PRES diagnosis in appropriate clinical settings.

## Introduction

Haematopoietic stem cell transplantation (HSCT) refers to intravenous infusion of haematopoietic progenitor cells derived from bone marrow, umbilical cord blood or peripheral blood to restore the haematologic and immunologic functions of bone marrow. In autologous transplantation (AutoHSCT), the donor is the patient himself/herself before bone marrow ablation. On the other hand, in allogeneic transplantation (AlloHSCT), the donor is usually human leukocyte antigen (HLA)-compatible or may sometimes be a haploidentical (half matched, mismatched) donor. HSCT is used worldwide in many malignant and nonmalignant childhood diseases, including haematologic, genetic, autoimmune and metabolic disorders. The survival rates of these diseases have been increased with HSCT treatment [1, 2]. However, associated complications are still important for management, which can be related to chemotherapy- or radiotherapy-related toxicity or can be associated with the state of immunosuppression predisposing patients to infection. Before HSCT, some recipients are prepared with high doses of chemotherapy and frequently with accompanying total body irradiation (TBI). These regimens may be myeloablative or lower-intensity nonmyeloablative. After HSCT, a chemotherapy regimen is initiated to prevent graft rejection and graft versus host disease (GVHD). Central nervous system (CNS) complications in paediatric HSCT recipients can be associated with high morbidity and significantly contribute to mortality. Moreover, the presence of neuroimaging abnormalities is associated with higher mortality in paediatric HSCT recipients [3]. One of the most common CNS complications in patients with neurological symptoms following HSCT is posterior reversible encephalopathy syndrome (PRES). In PRES, magnetic resonance imaging (MRI) is the modality of choice and shows typical bilateral vasogenic oedema. The most common presentation is posterior encephalopathy in which lesions are located at the posterior parts of the cerebral hemispheres, namely, the occipitoparietal lobes [4–6]. However, it may also show a nonposterior distribution and can be associated with various atypical imaging findings. In this study, we retrospectively examined CNS complications that occurred in our paediatric HSCT recipients who presented with CNS signs and symptoms with a focus on the typical and atypical neuroimaging findings of PRES following HSCT.

## Methods

We retrospectively reviewed the medical records of 300 consecutive paediatric patients who underwent HSCT from January 2014 to November 2018. A total of 130 paediatric HSCT recipients who experienced neurological signs and symptoms and were evaluated with neuroimaging studies following HSCT were enrolled in the study and the following data of 23 patients who had CNS complications were recorded: patient demographics, indications for and types of HSCT, pre- and post-transplant drug regimens, the application of total body irradiation (TBI), the presenting neurological signs and symptoms, and neuroimaging findings.Imaging studies, including magnetic resonance (MR) imaging of the brain and/or computed tomography (CT) of the head, were retrospectively evaluated for the presence of any imaging abnormalities. CT was performed in 48 patients as a first-line imaging method to exclude haemorrhagic events. The CT images were obtained using 64 channel MDCT scanners (Philips Medical Systems, Brilliance 64, the Netherlands). MR imaging was performed in 96 cases with the Siemens 3 T MAGNETOM Skyra MRI scanner with a dedicated head coil. The pulse sequences were coronal FLAIR (TE/TR =125/10000 msec; TI = 2800 msec), axial T2 (TE/TR = 80/3000 msec), axial T1 (10/2000 msec), diffusion-weighted imaging (DWI) (TE/TR = 120/3500 msec) with apparent diffusion coefficient (ADC) maps and GRE (16/840 msec) sequences. In 14 cases, both CT and MRI were performed. In addition to pathological signal intensities on conventional MRI sequences (T1W, T2W and FLAIR), any pathological findings on DWI, GRE and contrast-enhanced T1W images were also recorded. The timing of CNS complications was defined according to immune status, including the pre-engraftment period (< 30 days after HSCT), the early postengraftment period (30–100 days after HSCT), and the late postengraftment period (> 100 days after HSCT), which were defined as phases 1, 2 and 3, respectively.

## Results

**Fig. 1:**
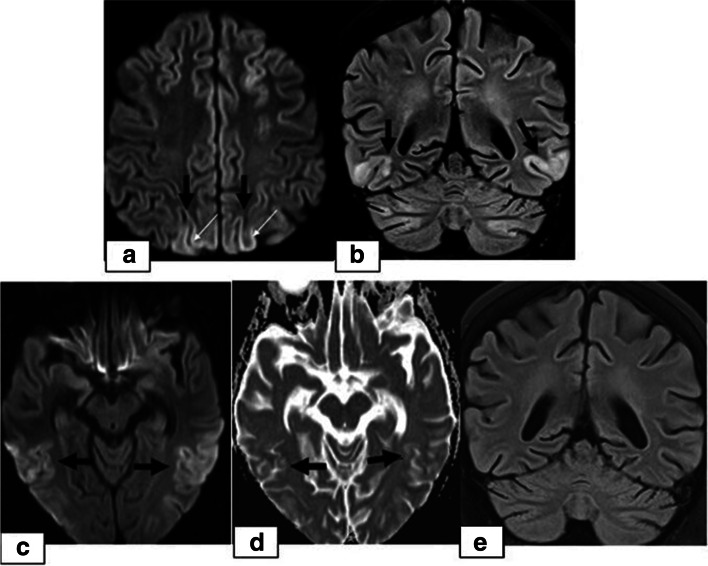
An 11-year-old boy presenting with seizures and visual disturbances 3 months after undergoing allogeneic HSCT for acute myeloid leukaemia. **a** Axial and **b**) coronal FLAIR images demonstrating bilateral symmetrical parietal (**a**) and occipital (**b**) cortico-subcortical hyperintensity **(black arrows)**. Note the prominent involvement of cortical grey matter **(1a, white arrow)**. **c** DWI and **d**) an ADC map showing a high signal **(arrows)** representing the T2 shine-through effect but not true restricted diffusion. **e** Follow-up coronal FLAIR image demonstrating complete resolution of vasogenic oedema in the occipital lobes after 3 months

**Fig. 2:**
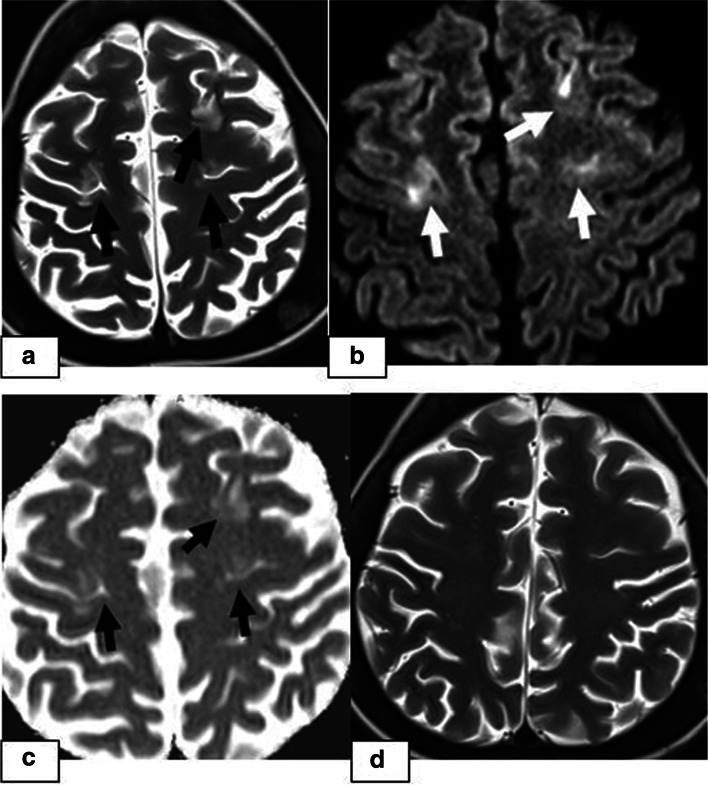
A 17-year-old girl presenting with an altered level of consciousness 6 months after undergoing allogeneic HSCT for thalassemia major. **a** Axial T2W image showing bilateral frontal cortico-subcortical hyperintensity **(arrows)**. **b** DWI and **c**) an ADC map showing a high signal **(arrows)** representing the T2 shine-through effect but not true restricted diffusion. **d** Axial T2W image demonstrating complete resolution of vasogenic oedema after 3 months

**Fig. 3:**
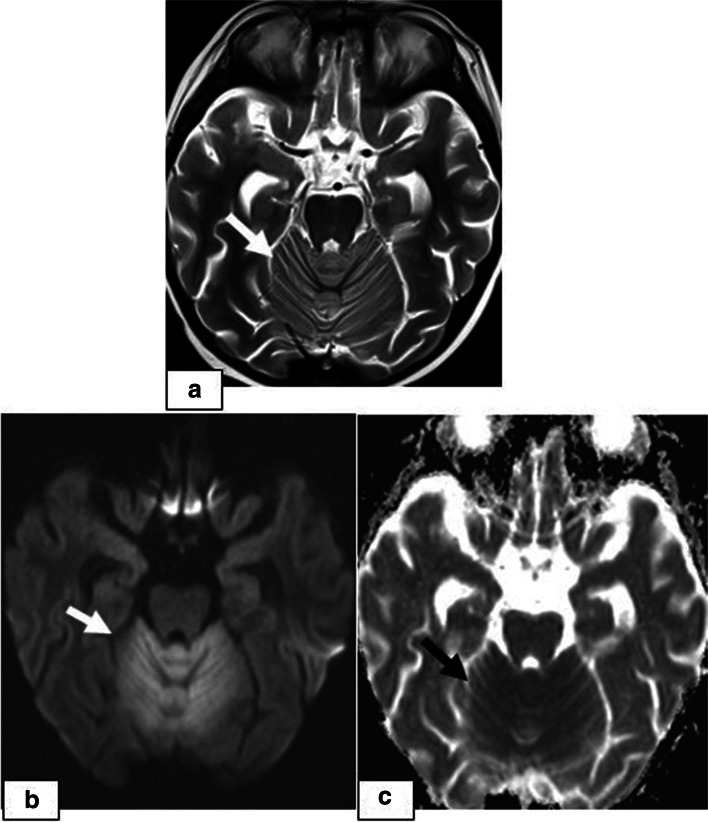
A 7-year-old girl presenting with seizures and headache 37 days after undergoing allogeneic HSCT for haemophagocytic lymphohistiocytosis. **a** Axial T2W image showing abnormal cerebellar hyperintensity **(arrows)**. **b** DWI and c) ADC images showing diffusion restriction **(arrows)**. She died 3 weeks after PRES onset due to acute pulmonary GVHD

**Fig. 4:**
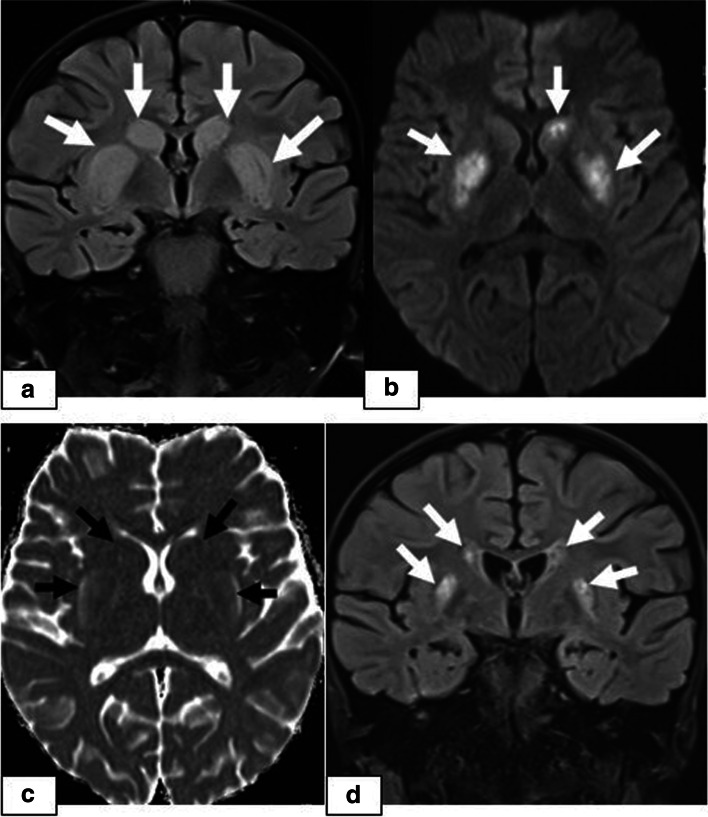
A 17-year-old girl presenting with an altered level of consciousness 6 months after undergoing allogeneic HSCT for thalassemia major. **a** Coronal FLAIR image showing hyperintensity in the bilateral basal ganglia **(arrows)**. **b** DWI showing increased signal intensity in the basal ganglia. **c** ADC map showing normal signal intensity, which was considered to be indicative of ADC pseudonormalization. **d** Follow-up coronal FLAIR image after 6 months demonstrating persistent hyperintensity associated with volume loss, suggesting cytotoxic injury

The study population included 130 paediatric HSCT recipients who experienced neurological signs and symptoms and therefore underwent neuroimaging examinations. Among these 130 patients, CNS complications were present in 23 patients (17.6%, 23/130). The patients included 13 (56.5%) females and 10 (43.5%) males with a median age of 8.0 years (range, 8 months to 18.0 years). The average age of the children at presentation was 8.40 ± 5.12 years. The underlying disorders for which HSCT was performed included acute lymphocytic leukaemia (ALL), acute myelogenous leukaemia (AML), lymphoma, thalassemia major, primary immune deficiencies, severe combined immune deficiency (SCID), Wiscott Aldrich syndrome (WA), haemophagocytic lymphohistiocytosis (HLH), sideroblastic anaemia (SA), aplastic anaemia (AA), Fanconi aplastic anaemia (FAA) and osteopetrosis. All of the patients underwent AlloHSCT. The sources of stem cells included bone marrow [*n* = 10], PBSCs [*n* = 9], bone marrow with PBSCs [*n* = 3] and bone marrow with cord blood [*n* = 1] from MSD [*n* = 4], MRD [*n* = 3], MUD [*n* = 10], and haploidentical donors [*n* = 6]. The preconditioning regimens were patient specific depending on the underlying disease and the types of donor and graft. All patients had received antibacterial, antifungal and antiviral prophylaxis. GVHD prophylaxis included calcineurin inhibitors, including cyclosporin A (CsA) (*n* = 18) and tacrolimus (*n* = 5) in combination with MTX with or without corticosteroids. The target serum concentration of CsA was determined to be 150–250 ng/mL for HSCT from MUT and haploidentical donors and 100–150 ng/mL for HSCT from MRT and MST donors. Five patients had received TBI (4 ALL, 1 lymphoma), and 7 patients had received chemotherapy (6 ALL, 1 AML) (Table 1). Twenty-three CNS complications included 14 cases of PRES (60.9%), 5 (21.7%) cases of leukoencephalopathy, 3 cases of acute subdural haemorrhage (SDH) (13%) and 1 (4.3%) case of fungal CNS infection (Tables 1, 2). The timing of the CNS complications is shown in Table 3. The most frequent neurological clinical signs and symptoms were headache followed by seizure, impaired consciousness and visual symptoms (Table 1). In PRES patients, the most frequent presentation was seizures, which were present in 71.4% of the PRES patients (10/14), followed by headache (50%) and visual symptoms (35,7%). While 6 cases of PRES occurred in phase 2, 5 cases were detected in phase 3, and 3 cases were detected in phase 1. Hypertension requiring antihypertensive treatment was present in 35.7% (5/14) of PRES cases at presentation. Of the 14 patients who developed PRES, 11 (78.5%) received cyclosporin A (CsA). In 4 (36.3%) of these cases, PRES developed when the CsA blood level was above the upper limit. In the remaining 7 (63.6%) patients, PRES occurred when the blood CsA level was within the normal range. Persistent signal changes were observed on follow-up MRI in 1 patient among the 4 patients who developed PRES and had a CsA blood level above the upper limit. Such changes were not observed in the patients whose CsA blood level was within the reference range, but this difference was not statistically significant (*p* > 0.05). On MRI, typical occipitoparietal vasogenic oedema was present in 78.5% of the PRES cases (11/14) (Table 4) (Fig. 1). The following atypical neuroimaging manifestations were identified: isolated involvement of bilateral frontal lobes in 1 case (Fig. 2), isolated cerebellar vermis involvement in 1 case (Fig. 3) and isolated basal ganglia involvement in 1 case (Fig. 4). MRI abnormalities were bilateral and almost symmetrical in all of the cases. Restricted diffusion associated with cytotoxic damage was demonstrated in 2 of 14 patients, one of whom had isolated cerebellar vermis involvement (Fig. 3). Unfortunately, she died 3 weeks after PRES onset due to acute pulmonary complications. The other patient presented with isolated basal ganglia involvement. In this patient, the lack of a dark signal on the ADC map despite increased signal intensity on DWI was considered ADC pseudonormalization rather than simple vasogenic oedema (Fig. 4). The median time of diagnosis from the day of first symptoms in PRES patients was 3 days for both typical and atypical presentations.While less delay was observed in the presentations with seizure and vomiting, the delay in diagnosis was more pronounced in patients with headache as an initial and dominant clinical finding. Follow-up imaging studies were performed in 9 of 14 patients with PRES. The other 5 patients died due to non-CNS complications. Among the 9 followed patients, reversibility was confirmed in 8 patients. In one patient with PRES who presented with basal ganglia involvement, persistent signal changes were detected 3 months after diagnosis. In this patient, the serum level of CsA was higher than the upper limit. In our patients with PRES, we did not detect haemorrhagic changes or pathologic contrast enhancement.

**Table 1:** Demographics of the 23 patients who experienced CNS complications

sex	age	diagnosis	type of donor	Chemot- herapy	Radio- therapy	GVHD prophylaxis	Signs and symptoms	CNS complications	Neuroimaging findings of PFES
Male	10	ALL	MUD	yes	no	MTX + CsA	seizure	PRES	Parieto-occipital involvement
Male	12	ALL	MUD	yes	no	MTX + CsA	headache	SDH	Unilateral left convexity
Female	5	MDS	Haplo.	no	no	MMF + TAC + CYC	impaired consciousness, headache	leukoencephalopathy	Symmetrical periventricular T2W/FLAIR hyperintensity
Female	0,75	SCID	Haplo.	no	no	MMF + TAC + CYC	Nausea- vomiting	SDH	Unilateral right frontoparietal
Female	9	AA	MRD	no	no	MTX + CsA	Headache, impaired consciousness,	Fungal infection	Multiple, small randomly distributed brain abscesses
Male	11	AML	Haplo.	yes	no	MMF + TAC + CYC	Seizure, Visual symptoms, headache	PRES	Parieto-occipital involvement
Female	7	HLH	Haplo.	no	no	MMF + TAC + CYC	Headache	PRES	Isolated Cerebellum involvement
Female	13	FAA	MUD	no	no	MTX + CsA	Seizure, Nausea- vomiting	PRES	Parieto-occipital involvement
Male	15	Lymphoma	MUD	no	TBI	MTX + CsA	Seizure, impaired consciousness	PRES	Parieto-occipital involvement
Female	8	ALL	Haplo.	yes	TBI	MTX + CsA + CYC	Nausea- vomiting	leukoencephalopathy	Symmetrical periventricular T2W/FLAIR hyperintensity
Female	4	SA	MUD	no	no	MTX + CsA	Headache, seizure	leukoencephalopathy	Symmetrical periventricular T2W/FLAIR hyperintensity
Male	7	ALL	MUD	yes	TBI	MTX + CsA	Headache, impaired consciousness	leukoencephalopathy	Symmetrical periventricular T2W/FLAIR hyperintensity
Female	4	TM	MSD	no	no	MTX + CsA	Headache, Seizure, visual symptoms	PRES	Parieto-occipital involvement
Female	8	TM	MSD	no	no	MTX + CsA	seizure	PRES	Isolated basal ganglia involvement
Female	3	TM	MUD	no	no	MTX + CsA	Visual symptoms, headache	PRES	Parieto-occipital involvement
Male	0,66	Osteopetrosis	MRD	no	no	MTX + CsA	Nausea- vomiting	SDH	Unilateral left frontoparietal
Male	0,91	WA	Haplo.	no	no	MMF + TAC + CYC	Seizure	PRES	Parieto-occipital involvement
Female	17	TM	MRD	no	no	MTX + CsA	Headache, Nausea- vomiting	PRES	İsolated frontal involvement
Female	5	TM	MUD	no	no	MTX + CsA	Headache, seizure, impaired consciousness	PRES	Parieto-occipital involvement
Male	14	ALL	MUD	yes	TBI	MTX + CsA	seizure	PRES	Parieto-occipital involvement
Male	13	TM	MSD	no	no	MTX + CsA	Visual symptoms, Nausea- vomiting, seizure	PRES	Parieto-occipital involvement
Female	18	TM	MUD	no	no	MTX + CsA	impaired consciousness, visual symptoms	PRES	Parieto-occipital involvement
Male	8	ALL	MSD	yes	TBI	MTX + CsA	impaired consciousness, headache	leukoencephalopathy	Symmetrical periventricular T2W/FLAIR hyperintensity

CsA: CYC: MMF: MTX: TAC:

**Table 2:** CNS Complications

	n	%
Leukoencephalopathy	5	21,7
PRES	14	60.9
Fungal CNS infection	1	4.3
SDH	3	13

**Table 3:** Twenty-three CNS complications following HSCT in relation to the chronology of HSCT

	Phase 1	Phase 2	Phase 3	Number
PRES	3	6	5	14
Leukodystrophy		1	4	5
Acute Subdural haemorrhage	3			3
Fungal CNS infection			1	1

**Table 4:** Encephalic locations in 14 PRES cases

Involvement of encephalic locations	%
Typical occipitoparietal involvement	78.5%
Atypical involvement	21.5%
-isolated bilateral frontal lobes	7,16%
-isolated basal ganglia	7,16%
-isolated cerebellar vermis	7,16%

**Fig. 5:**
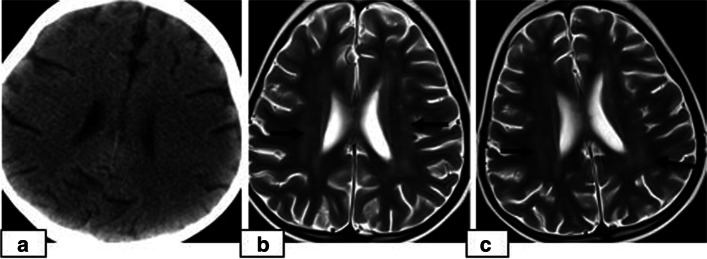
An 8-year-old girl presenting with headache, nausea and vomiting 5 months after undergoing allogeneic HSCT for ALL. **a** Axial CT of the head did not reveal any abnormalities. **b** Axial T2W image showing increased signal intensity in the periventricular white matter (b, arrows). **d** Follow-up axial T2W image 3 months after the first presentation shows persistent increased signal intensity in the periventricular white matter (c, arrows)

**Fig. 6:**
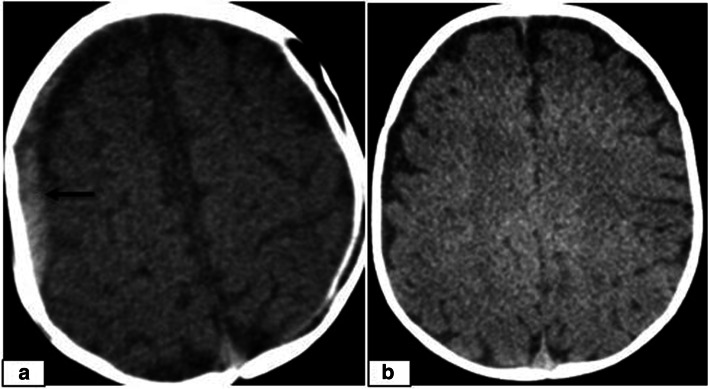
A 9-month-old female girl presenting with nausea and vomiting 25 days after undergoing allogeneic HSCT for SCID. **a** Axial CT image showing acute hyperdense right frontoparietal SDH **(arrow)**. b Follow-up axial CT image 1 month after the onset of acute SDH showing complete resolution

**Fig. 7:**
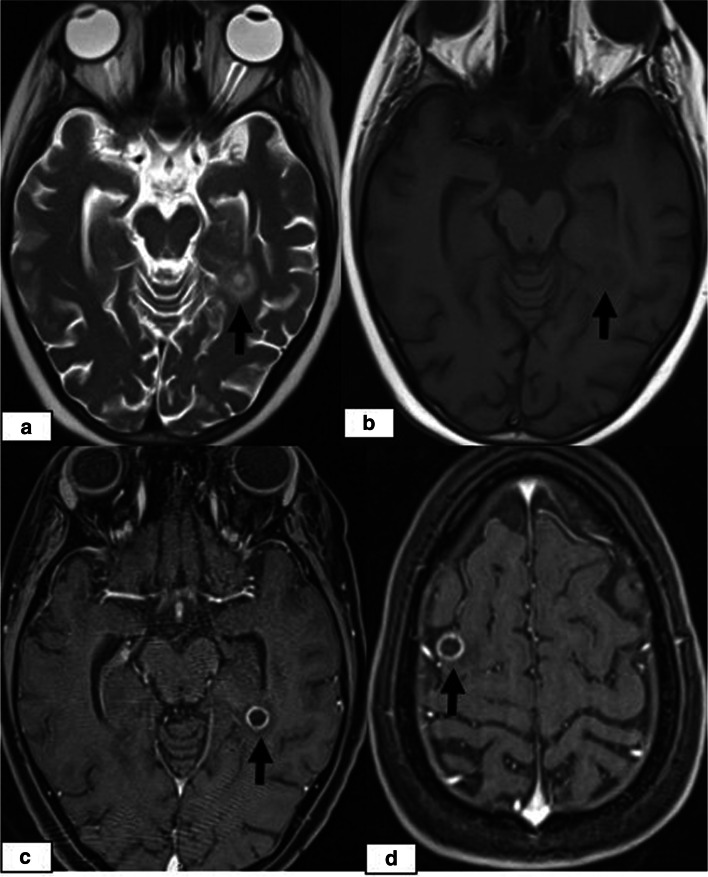
A 9-year-old girl presenting with headache and impaired consciousness 5 months after undergoing allogeneic HSCT for aplastic anaemia. **a** Axial T2W image showing a cystic lesion with peripheral oedema in the left medial temporal region **(arrow)**. **b** Axial T1w image showing hypointensity of the lesion. **c**, **d** Postcontrast axial T1W images showing ring enhancement of the same lesion and another similar lesion at the frontal convexity (d, arrow) compatible with a brain abscess

Leukoencephalopathy was identified in 5 patients, which occurred in one patient during phase 2 and in the remaining patients during phase 3 (Table 3). On MRI, periventricular white matter hyperintensity on T2/FLAIR images with no diffusion restriction was present with normal preceding CT images. In the follow-up, total resolution of the MRI signal abnormalities was not observed in any of the cases (Fig. 5). Intracranial haemorrhage was identified in only 3 cases, all of which were acute SDH occurring in phase 1, and in the follow-up of these patients, total haemorrhage resolution was confirmed (Fig. 6). Fungal CNS infection was observed in one of the patients with a positive sputum culture for *Aspergillus* in the intensive care unit (ICU) (Fig. 7).

## Discussion

The incidence of CNS complications following HSCT varies considerably in different studies depending on the patients’ demographic and clinical data, including pre-post-transplant drug regimens, the application of TBI, the degree of immune suppression or the development of GVHD. The rate is higher in patients with AlloHSCT than in patients with AutoHSCT and reaches up to 70% in some studies [7, 8]. In a recently conducted study by Hussein et al., which retrospectively evaluated 525 HSCT recipients, the prevalence was reported to be 13% [9]. In our study, the prevalence was 7.66% (23/300), which is similar to the rate of 8.67% (26/300) reported by Suxiang Liu et al. [10]. The prevalence among the recipients presenting with CNS signs and symptoms following HSCT in our study was 17.6% (23/130). Among these 23 patients, the most common clinical signs and symptoms were headache followed by seizure, visual symptoms and impaired consciousness. In our study, PRES was the most common CNS complication, which was observed in 4.6% of the 300 HSCT recipients (14/300) and was more frequent at < 100 days post-HSCT. Although different causes are responsible for PRES in paediatric patients, it is mostly described as a complication following various types of transplantation, and in many studies, it is the most common neuroimaging abnormality following HSCT. The incidence of PRES following allogenic HSCT in paediatric patients varies between 1.1–34% in different clinical studies in the literature and is affected by various factors, including the drugs used in the conditioning regimens and GVHD prophylaxis, the presence of HT, the level of immune suppression, underlying diseases, the type of transplantation, and the presence of triggering factors such as infections and GVHD [4, 11–13]. In a study retrospectively evaluating 35 paediatric HSCT recipients, the incidence of PRES was reported to be 17% (*n* = 6). In this study, all PRES patients were taking CNIs at the time of symptom onset, and the median time after HSCT to PRES onset was 21 days (phase 1) [14]. PRES is a clinical and radiologic diagnosis characterized by variable presentations with various combinations of acute neurological symptoms. In paediatric patients diagnosed with PRES, the most frequently reported primary presentation is seizures, as in our PRES patients [15]. The underlying pathophysiologic mechanism is controversial, and two main theories have been proposed. According to vasogenic theory, high blood pressure causes dysregulation of cerebrovascular autoregulation, resulting in cerebral vasodilation and oedema [5, 16]. However, arterial hypertension is not present in all patients with PRES. In our study, hypertension requiring antihypertensive treatment was present in 5 cases of PRES. On the other hand, according to cytotoxic theory, the cause is increased microvascular permeability as a result of direct toxic effects on endothelial cells [17]. The absence of increased blood pressure in many of the patients supports the cytotoxic theory, as in observed our cases. Immunosuppressive medications, such as CsA, TAC, and steroids, which are the most commonly used drugs for GVHD prophylaxis, can induce PRES in HSCT recipients [18–20]. On MRI, typical findings of cerebral vasogenic oedema as a result of extravasation of plasma proteins and cells into the extracellular space are demonstrated. In our patients, typical occipitoparietal vasogenic oedema was present in 78.5% of the PRES cases (11/14) (Fig. 1). In many studies, occipitoparietal involvement was predominantly reported to vary between 50 and 99% of their cases [15, 20]. Cerebral cortical (grey matter) involvement is observed in many patients, as in our cases [21, 22]. (Figs. 1 and 2). Despite being termed posterior, PRES can also show other distributions, mainly in watershed areas, which can be involved in combination or in isolation [23]. The uncommon locations observed in our patients were as follows: isolated involvement of the frontal lobes (Fig. 2), cerebellum (Fig. 3) and basal ganglia (Fig. 4). The term central PRES is used to describe isolated involvement of the basal ganglia, thalamus, brain stem and corpus callosum with a lack of cortico-subcortical involvement. The central variant of PRES was reported in 4% of cases in the study of McKinney et al. [24]. In the study of Raman et al. [25], the basal ganglia were involved in 22%, the brainstem in 9% and the thalamus in 4% of the cases. However, all of these cases also had lesions in the bilateral parietooccipital subcortical white matter. In our study, a central PRES variant with isolated involvement of the basal ganglia was observed in 1 patient (Fig. 4). In PRES, the lesions are usually symmetrical, as in our cases. However, purely unilateral cases of PRES have also been demonstrated in the literature [16, 24]. The symmetrical involvement and reversibility of the MRI findings in the patient with frontal involvement was compatible with PRES. In the patient with isolated basal ganglia and cerebellar vermis involvement presenting with restricted diffusion and the patient exhibiting the symmetrical basal ganglia involvement and restricted diffusion only in the basal ganglia and the vermis without extension to the cerebellar hemispheres, the findings suggested toxic metabolic aetiologies rather than vascular pathology. We excluded all other toxic metabolic aetiologies, including metabolic toxins (such as carbon monoxide), hypoglycaemia, hyperammonemia or hypoxia.

In PRES lesions, increased ADC values are characteristic and indicative of vasogenic oedema. DWI may be normal, or hyperintensity is often observed due to the T2 shine-through effect. However, true restricted diffusion may also present as an atypical finding in PRES lesions [26], which is important because higher ADC values are associated with reversibility, while decreased ADC values indicate cytotoxic injury and a poor prognosis [27]. In the study of McKinney et al. [24], 17.3% of the 76 patients with PRES had restricted diffusion, and in the study of Covarrubias et al. [28], 27% of 22 patients with PRES showed restricted diffusion. In the study of Hussein et al., the incidence of PRES in post-HSCT recipients was 3.2%, with the most frequent sites being the occipital and parietal regions in 88.2 and 82.4% of the patients, respectively. In their study, diffusion restriction was observed in 29.4% of the cases (*n* = 5), and no significant dark signal on ADC maps associated with pseudonormalization was noted in any of the 5 cases [9]. In our study, restricted diffusion was demonstrated in 2 of 14 PRES patients, one of whom showed isolated cerebellar vermiş involvement. Unfortunately, since the patient died from acute pulmonary complications 3 weeks after the onset of PRES, no follow-up MRI was available to determine whether the abnormal signals persisted (Fig. 3). The other patient presented with basal ganglia involvement, and an increased DWI signal was accompanied by normal ADC values indicating ADC pseudonormalization, which is a normal phase in the subacute stage of cytotoxic injury [29]. In the follow-up imaging studies of this patient, whose diagnosis was delayed, the latency period was characterized by volume loss and persistent signal changes, which is consistent with the association of restricted diffusion with persistent changes and a poor prognosis (Fig. 4).

The other reported atypical neuroimaging findings associated with PRES is accompanying haemorrhage and contrast enhancement, which were not present in our cases. Although PRES, as its names implies, is mostly reversible, residual sequelae formation can occur. In the follow-up, clinical recovery is usually observed earlier than disappearance of imaging findings and is usually associated with a good clinical outcome with early diagnosis and management.

The incidence of post-HSCT leukoencephalopathy was 1.6% in our study and was reported to be 1.9% in the study of Hussein S.A. et al. [14]. Four of our patients had CsA and MTX in their anti-GVHD regimen, 3 of whom had received TBI. In all 5 patients, CT images did not demonstrate any abnormality due to isodensity of the involved white matter, requiring further MRI studies to reveal hyperintensity on T2/FLAIR images with no diffusion restriction. In the follow-up images, a stable course was observed in all 5 cases; however, total resolution of the MRI signal abnormalities was not observed in any of the cases in our study (Fig. 5). Among our patients, intracranial haemorrhage was present in 3 patients, all of whom had acute SDH, and in the follow-up of these patients, total resolution of the haemorrhage was confirmed (Fig. 6). In the study of Hussein et al., the incidence of intracranial haemorrhage was reported to be 1.5% (*n* = 8), with 3/8 (37.5%) being SDH (36). We observed only one case of CNS infection, which occurred when the patient was followed in the intensive care unit, and multifocal invasive *Aspergillosis* was confirmed by sputum culture. MRI showed multiple randomly distributed enhanced rings surrounding small brain abscesses. Unfortunately, this patient died after a short time, and no CSF or histopathological confirmation was obtained prior to her death (Fig. 7).

## Conclusion

Paediatric HSCT recipients presenting with CNS signs and symptoms should be evaluated by neuroimaging studies for timely diagnosis and early management. PRES is the most common CNS complication and usually presents with typical clinical and imaging findings. However, atypical MRI manifestations may appear, which should not dissuade a PRES diagnosis in appropriate clinical settings with exclusion of other possibilities. Diffusion restriction is an important imaging finding that can be associated with residual changes and should be assessed in all MRI studies for these patients.

### Main points

* Paediatric HSCT recipients presenting with CNS signs and symptoms should be evaluated by neuroimaging studies.

* Variable neuroimaging presentations should not dissuade a PRES diagnosis in appropriate clinical settings following allogeneic HSCT in paediatric recipients.

* In PRES, lower ADC values are associated with a poor prognosis, and normal ADC values in association with increased signal intensity on DWI should also be evaluated in terms of ADC pseudonormalization.

## Data Availability

The data sets used and/or analysed during the current study are available from the corresponding author upon reasonable request.
